# Impact of Dietary Protein Restriction on the Immunogenicity and Efficacy of Whole-Sporozoite Malaria Vaccination

**DOI:** 10.3389/fimmu.2022.869757

**Published:** 2022-04-21

**Authors:** Helena Nunes-Cabaço, Diana Moita, Catarina Rôla, António M. Mendes, Miguel Prudêncio

**Affiliations:** Instituto de Medicina Molecular João Lobo Antunes, Faculdade de Medicina, Universidade de Lisboa, Lisbon, Portugal

**Keywords:** malaria, vaccination, nutrition, dietary protein, immune response

## Abstract

Malaria remains one of the world’s most prevalent infectious diseases. Several vaccination strategies currently under investigation aim at hampering the development of the *Plasmodium* parasite during the clinically silent liver stage of its life cycle in the mammalian host, preventing the subsequent disease-associated blood stage of infection. Immunization with radiation-attenuated sporozoites (RAS), the liver-infecting parasite forms, can induce sterile protection against malaria. However, the efficacy of vaccine candidates in malaria-naïve individuals in high-income countries is frequently higher than that found in populations where malaria is endemic. Malnutrition has been associated with immune dysfunction and with a delay or impairment of the immune response to some vaccines. Since vaccine efficacy depends on the generation of competent immune responses, and malaria-endemic regions are often associated with malnutrition, we hypothesized that an inadequate host nutritional status, specifically resulting from a reduction in dietary protein, could impact on the establishment of an efficient anti-malarial immune response. We developed a model of RAS immunization under low protein diet to investigate the impact of a reduced host protein intake on the immunogenicity and protective efficacy of this vaccine. Our analysis of the circulating and tissue-associated immune compartments revealed that a reduction in dietary protein intake during immunization resulted in a decrease in the frequency of circulating CD4+ T cells and of hepatic NK cells. Nevertheless, the profile of CD8+ T cells in the blood, liver and spleen was robust and minimally affected by the dietary protein content during RAS immunization, as assessed by supervised and in-depth unsupervised X-shift clustering analysis. Although mice immunized under low protein diet presented higher parasite liver load upon challenge than those immunized under adequate protein intake, the two groups displayed similar levels of protection from disease. Overall, our data indicate that dietary protein reduction may have minimal impact on the immunogenicity and efficacy of RAS-based malaria vaccination. Importantly, this experimental model can be extended to assess the impact of other nutrient imbalances and immunization strategies, towards the refinement of future translational interventions that improve vaccine efficacy in malnourished individuals.

## Introduction

Malaria, a mosquito-borne disease caused by *Plasmodium* parasites, remains one of the world’s most prevalent infectious diseases, with 241 million cases reported worldwide in 2020, and children under 5 years old representing 77% of the estimated 627,000 malaria fatalities (1). There is currently intense research dedicated to the development of much-needed vaccines that can prevent malaria and its devastating effects.

Vaccination strategies aim at halting the development of the *Plasmodium* parasite at different stages of its complex life cycle, which involves both mammalian and mosquito hosts. The most efficient vaccine candidates take advantage of the obligatory but clinically silent liver stage of *Plasmodium* infection, during which sporozoites, the parasite form injected into the host’s skin by *Anopheles* mosquitoes, infect and develop inside hepatocytes before egressing to the blood, where they infect erythrocytes and cause disease symptoms (2, 3). Whole-sporozoite (Wsp)-based vaccination represents a highly effective pre-erythrocytic immunization strategy (4). *P. falciparum* (*Pf*)-based Wsp vaccine candidates developed thus far employ either radiation-attenuated sporozoites (RAS), genetically attenuated sporozoites (GAS) or live sporozoites in combination with chemoprophylaxis (CPS) (2–6) as immunization agents. Recently, an alternative strategy based on genetically modified *P. berghei* (*Pb*) rodent malaria parasites expressing the highly immunogenic *Pf* circumsporozoite protein (PbVac) was also developed (7, 8) and clinically validated (9).

Vaccination with RAS induces sterile protection against malaria in mice, non-human primates and humans, and is considered the gold-standard of Wsp vaccination (2–6, 10). RAS immunization induces potent local liver immunity, centrally mediated by non-recirculating CD8+ resident-memory T (T_RM_) cells that patrol the liver sinusoids for continuous pathogen surveillance and can be harnessed to control liver infection (11–14). In clinical trials involving *Pf*SPZ, a vaccine candidate that employs aseptically purified, cryopreserved *Pf*RAS, less than 30% of protection was found in individuals in Mali (15) or Tanzania (16) as compared to 65-100% in the USA. Although the failure to provide adequate protection in individuals of African countries could be at least partially explained by immunoregulation caused by previous exposure to *Plasmodium* antigens, other factors may contribute to the decreased protective efficacy observed.

People in greater need of protection against malaria, such as those living in Sub-Saharan Africa and Southeast Asia, also likely endure malnutrition, especially during the annual rainy season. Malnutrition is a global health issue, affecting more than 200 million children under 5 years of age worldwide, and the underlying cause of 45% of deaths in children of that age (17, 18). It commonly involves an intake of macronutrients (protein, carbohydrates, fat), micronutrients (minerals, vitamins, electrolytes), or both, that is inadequate to meet the dietary requirements of the body and ensure the most favorable growth (17–19). Malnutrition has been associated with innate and adaptive immune dysfunction, leading to increased susceptibility to infection and disease and to a delay or impairment in the immune response to some vaccines (13, 15–17).

Protein deficiency is commonly observed in developing countries (13–15, 17, 18). According to statistics from the Food and Agriculture Organization of the United Nations (FAO), the average protein supply quantity in 2018 in Europe and North America was 102,72 and 112,76 g/capita/day, respectively, while it was at least 30-40% lower in Africa and Southeast Asia (68,11 and 73,19 g/capita/day, respectively) (20). Severe protein-energy malnutrition (PEM) has been associated with spleen and thymic atrophy and a decreased number of B and T cells, including CD4+ helper (mainly Th1) and CD8+ T lymphocytes, as well as with an impact on memory CD8+ T cell function (21–26).

The effect of protein deficiency on *Plasmodium* infection in humans is controversial, with some studies associating PEM with increased morbidity and others with a protective effect (19, 27–29). Studies in non-human primates and murine models of malaria showed an impact of low protein diets on *Plasmodium* infection, including suppression of parasitemia and protection against rodent experimental cerebral malaria, as well as an inability to clear the infection and, in some cases, increased mortality (19, 27, 30–32).

Since vaccine efficacy depends on competent humoral and cellular immune responses, and relies heavily on long-lived memory formation, we hypothesized that the effect of a low protein diet on the immune compartment might have an impact on the efficacy of malaria vaccination. We thus established a RAS immunization protocol that allowed the assessment of the effect of low dietary protein, comparable to that observed in malaria-endemic countries, on the immunogenicity and efficacy of malaria vaccination. Since protein deficiency may affect malaria infection (19, 27–32), our protocol was designed to minimize the potential impact of the nutritional imbalance on parasite development. Our extensive characterization of the circulating and tissue-associated immune compartments revealed that immunization under a low protein diet led to reduced circulating CD4+ T cell and hepatic NK cell frequencies, although most immune alterations, namely in the CD8+ T cell population, were related to the immunization *per se*. Importantly, despite the increased parasite liver load in mice immunized under reduced protein intake, we found no diet-associated differences in protection against *Plasmodium* infection, supporting the efficacy of RAS malaria vaccination in low protein settings. This protocol can be extended to other immunization and nutritional settings, aiding the rationale for nutritional supplementation strategies in the context of malaria vaccination.

## Materials And Methods

### Animals and Diets

Male C57BL/6J mice (5-6 weeks old) were purchased from Charles River Laboratories (Lyon, France) and housed under specific pathogen-free (SPF) conditions at the rodent facility of iMM-JLA (licensed under the European Directive 2010/63/EU on the protection of animals used for scientific purposes). The study was approved by iMM-JLA’s animal ethics committee (ORBEA) and all experimental animal work was performed in strict compliance with the guidelines of the Federation of European Laboratory Animal Science Associations (FELASA), particularly the 3 Rs (Replacement, Reduction, Refinement). Mice were housed in climate-controlled conditions (25°C, 12h light/dark photoperiod) and were maintained under adequate (20%; Ref. C1000) or low (9%; Ref. C1003) protein diets (from Altromin International, Lage, Germany), with food and water *ad libitum*. The decrease in dietary protein was compensated with an increase in carbohydrates to maintain isocaloric diets of ~3500 kcal/kg. Individual mouse weight and food/water consumption per cage were monitored 1-3 times per week.

### Immunization and Challenge With *P. berghei* Parasites

Luciferase-expressing *P. berghei* ANKA sporozoites (33, 34) were obtained through dissection of salivary glands from infected female *Anopheles stephensi* mosquitoes reared at iMM-JLA. Radiation-attenuated sporozoites (RAS) were obtained through exposure of freshly dissected sporozoites to γ-radiation (16,000 rad in a Gammacell 3000 ELAN irradiator). Ten thousand sporozoites or RAS were inoculated into mice by retro-orbital intravenous (i.v.) injection, according to the schedule described. Re-challenges were performed 2 or 4 months after the first challenge using 10,000 infectious *Pb* sporozoites.

### Plasma Biochemical Parameters

Blood was drawn by cardiac puncture into heparinized syringes and centrifuged for 10 minutes at 2000 g. The following parameters were determined in the plasma (DNAtech, Lisbon, Portugal): total protein, albumin, glucose, alanine aminotransferase (ALT) and blood urea nitrogen (BUN).

### Determination of Blood Parasitemia and of *In Vivo* Liver Infection

*P. berghei* parasitemia was determined in blood collected from the mouse tail using the Firefly Luciferase Assay Kit 2.0 (Biotium, Fremont, CA, USA), according to the manufacturer’s instructions. *In vivo* liver infection was quantified by imaging using XenoLight D-Luciferin - Potassium Salt Bioluminescent Substrate (PerkinElmer, Waltham, MA, USA) and the IVIS Lumina Imaging System (Caliper LifeSciences, Waltham, MA, USA).

### Blood and Tissue Processing

Analysis of circulating immune populations was performed in 10 µl of blood collected from the mouse tail into 90 µl of PBS with 10 U/ml heparin. After centrifugation for 5 minutes at 600 g the diluted plasma was removed and placed at -80 °C, while the pelleted cells were treated with red blood cell ammonium–chloride–potassium lysis buffer (ACK) for 5 minutes, washed with 2% fetal bovine serum (FBS) in PBS (FACS buffer) and analyzed by flow cytometry. For tissue analysis, mice were extensively perfused in the left ventricle with PBS and the liver and spleen were removed. One liver lobe was snap frozen in liquid nitrogen and kept at -80 °C until RNA extraction. The remaining liver and the spleen were processed for flow cytometry analysis, as described (35). Briefly, the tissues were mashed in FACS buffer and treated with ACK, with liver leukocytes separation involving an additional Percoll gradient step before ACK treatment.

### Flow Cytometry Analysis

Total blood cells or leukocytes from the liver or spleen were incubated with Fc block (eBiociences, Thermo Fisher Scientific) for 10 minutes at 4-8°C. Surface staining was performed for 20 minutes at 4-8°C and always included Fixable Viability Dye (eBioscience, Thermo Fisher Scientific, Waltham, MA, USA) for exclusion of dead cells. The following conjugated anti-mouse mAbs (and respective clones) were used: CD3 PerCP-Cy5.5 (145-2C11), CD4 APC or Brilliant Violet (BV) 510 (GK1.5), CD8 BV711 (53-6.7), CD62L FITC (MEL-14), CD19 PE (1D3), CD25 APC (3C7), CD44 PE-Cy7 (IM7), CD45 Alexa Fluor 700 (30-F11), CD69 BV650 (H1.2F3), CD127 PE-Dazzle 594 (A7R34), NK1.1 PE-Cy5 (PK136), KLRG1 PE (MAFA), CXCR3 APC (CXCR3-173), TCR γδ BV421 (GL3), all from either BioLegend (San Diego, CA, USA) or Sysmex (Kōbe, Hyōgo, Japan). Cells were acquired in a BD LSR Fortessa X-20 cytometer and analyses were performed within live, single (based on FSC-A vs. FSC-W parameters) CD45+ leukocytes using FlowJo v10 (FlowJo, BD). For the clustering and visualization of high-dimensional data, equivalent numbers of live, single CD45+ cells from each condition were concatenated. Clustering was performed using X-shift (number of clusters determined by the algorithm) and data are presented using the dimensionality reduction method TriMAP (large-scale dimensionality reduction using triplets).

### Quantification of Liver Genes by RT-qPCR

Total RNA was purified from the liver using the TripleXtractor direct RNA kit (Grisp), according to the manufacturer’s instructions. cDNA was synthesized from 1 µg of RNA using random primers and the NZYTech cDNA synthesis kit (NZYTech, Lisbon, Portugal). Quantification was performed with NZYSpeedy qPCR Green Master Mix (2x), ROX (NZY Tech, Lisbon, Portugal) using an Applied Biosystems ViiA7 Real-Time PCR System (Applied Biosystems, Foster City, CA, USA). Sterol regulatory element binding transcription factor 1 (*srebf1*) RNA was quantified using predesigned KiCqStart^®^ SYBR^®^ Green Primers (M_Srebf1_1; Sigma-Aldrich). For the other genes the following primers (forward/reverse) were used: fatty acid synthase (*fas)*: GGAGGTGGTGATAGCCGGTAT/TGGGTAATCCATAGAGCCCAG; hypoxanthine-guanine phosphoribosyl transferase (*Hprt*;reference gene): TTTGCTGACCTGCTGGATTAC/CAAGACATTCTTTCCAGTTAAAGTTG; *P. berghei 18S rRNA*: AAGCATTAAATAAAGCGAATACATCCTTAC/GGAGATTGGTTTTGACGTTTATGTG.

### Assessment of the Humoral Response to *P. berghei*

Circulating IgG antibodies against *P. berghei* sporozoites were measured in the plasma by ELISA, according to previously described protocols (8, 9) with some modifications. Briefly, purified, frozen *P. berghei* sporozoites were thawed and treated with an extraction buffer (150 mM NaCl, 20 mM Tris-HCl, 1% triton, 1 mM EDTA, pH= 7.5) for 15 minutes at 4°C. ELISA plates (Nunc MaxiSorb) were coated overnight at 4°C with lysates corresponding to 5,000 *P. berghei* sporozoites per well, washed with 0,05% Tween20 in PBS (PBST) and incubated in blocking buffer (5% nonfat dry milk in PBST) for 1 h at room temperature. Diluted plasma samples or control antibodies (anti-*Pb*CS 3D11 mAb) were added to each well for 2 h at room temperature. After washing the wells with PBST, an HRP-conjugated goat anti-mouse IgG secondary antibody (sc-2005, Santa Cruz Biotechnology, Dallas TX, USA) was added at 1:2000 dilution for 1h at room temperature. Plates were washed and developed using a TMB Substrate Reagent Set (BD OptEIA, BD Biosciences), according to the manufacturer’s protocol. The reaction was stopped after 5-10 min with 1N H_2_S0_4_ and absorbance was measured at an optical density (OD) of 450 and 570 nm (reference wavelength) using a TECAN Infinite M200 microplate reader.

### Determination of IFN-γ in the Blood

The concentration of IFN-γ in the plasma was determined using the LEGEND MAX™ Mouse IFN-γ ELISA Kit (BioLegend), according to the manufacturer’s instructions. Absorbance was measured at an OD of 450 and 570 nm (reference wavelength) using a TECAN Infinite M200 microplate reader.

### Statistical Analysis

Statistical analysis was performed using GraphPad Prism v9 (GraphPad Software Inc.) and results are presented as mean ± SD. Relationships between two variables were evaluated using nonparametric Spearman’s rank correlation coefficient. Two-sample data were compared using Mann-Whitney test. Data from more than two samples were compared using Kruskal-Wallis test (One-Way ANOVA) with Dunn’s multiple comparison post-test. Data was considered statistically significant when P (or adjusted P) value was <0,05 and represented as: *: 0,05>P>0,01; **: 0,01>P>0,001; ***: 0,001>P>0,0001; ****: P<0,0001.

## Results

### Establishment of a Low Protein Diet Protocol That Specifically Targets the Host, Without Impacting on Plasmodium Infection

Studies on protein reduction or PEM commonly employ protein-deficient or nearly deficient diets, which correspond to a decrease of more than 80% of the dietary protein content. Although this may mimic the situation observed in some geographical areas, the average protein supply in malaria-endemic regions, such as Africa and Southeast Asia, is estimated to be approximately 65-70% of that observed in the USA or Europe (20), where many clinical trials of malaria vaccine candidates take place. It is possible that this may constitute an overestimation, since this calculation does not consider the correction for household losses (estimated at 25–30%), and protein consumption in the USA and Europe is well above the world average (20). To develop a model that more closely mimics protein reduction in malaria-endemic regions, we established a protocol where the protein dietary content was approximately 45% of that included in a regular diet.

**Figure 1:**
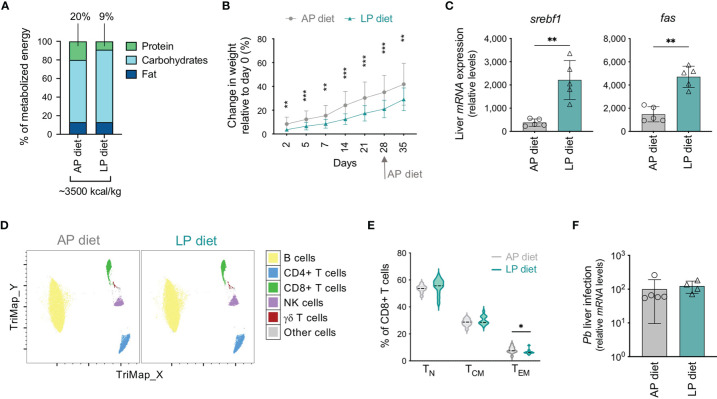
Optimization of a protocol of low protein diet that impacts the host but does not influence parasite development. **(A)** Composition of the adequate (AP, 20%) or low (LP, 9%) protein diets used in the study. **(B)** Change in weight (relative to day 0) of mice under AP or LP diet. Data were from 16-18 mice of 3 independent experiments. **(C)** Hepatic expression of *srebf1* and *fas* on day 35 in mice maintained under each protocol. Values were normalized to *hprt* expression. Each symbol represents one mouse. **(D)** TriMap dimensionality reduction analysis of circulating immune populations in concatenated data of mice maintained under AP or LP diets on day 28, based on the following markers: CD3, CD4, CD8, CD19, CD25, TCRγδ and NK1.1. **(E)** Violin plots of the frequency of naïve (T_N_), central memory (T_CM_) and effector/effector memory (T_EM_) populations within CD8+ T cells in mice maintained under AP or LP diet, on day 28 (n = 11-13 per group). **(F)** *Pb* liver load at 48h post-infection (10,000 infectious *Pb* sporozoites) of mice maintained under each protocol and infected on day 35. Levels of hepatic *Pb* 18S rRNA were normalized to *hprt* expression. Data are presented as mean ± SD and were compared using the Mann-Whitney test (*P < 0,05; **P < 0,01; ***P < 0,001).

Mice were maintained under isocaloric adequate (AP, 20%) or low (LP, 9%) protein diets (**Figure 1A**) for 4 weeks, at which time-point all mice were placed on AP diet for one additional week. This diet transition was required in order to avoid the direct impact of protein reduction on parasite development upon infection. Although food consumption by mice under LP or AP diet was similar (**Figure S1A**), from day 2 onwards the weight gain of mice under LP diet was significantly lower than that of animals under AP diet (**Figure 1B**). Of note, water ingestion by mice on LP diet was increased relative to their counterparts under AP diet (**Figure S1A**). While no statistically significant alterations were found in plasma metabolites in the two groups of mice at the end of 5 weeks (**Figure S1B**), the altered hepatic expression of the lipogenesis-related molecules sterol regulatory element binding transcription factor 1 (*srebf1*) and fatty acid synthase (*fas*) is indicative of a clear effect of the diet on the host at this time-point (**Figure 1C**). These results validate an effect of the diet employed in this study on liver metabolism.

We analyzed circulating immune populations on the day of replacement of LP by AP diet using standard flow cytometry hierarchical gating strategy (**Figure S1C**) as well as TriMap dimensionality reduction analysis (**Figure 1D**) (33). No major changes were found in the overall unbiased immune profile (**Figure 1D**) or in the frequency or numbers (**Figures S1D, E**) of most populations analyzed, including B cells, CD4+ T, γδ T, regulatory T (Treg) and NK cells. Nevertheless, mice under LP diet displayed an increase in the frequency of CD8+ T cells (**Figure S1D**), but a decrease in the proportion of the effector/effector memory (T_EM_) subset (**Figure 1E**), relative to those under AP diet. Thus, and despite the relatively mild protein reduction in our model as compared to studies addressing severe malnutrition, alterations in the circulating CD8+ T cell population could be observed under the experimental conditions employed.

Protein deficiency was reported to impact on *Plasmodium* infection and to decrease blood parasitemia (27, 29). We confirmed that LP diet affected *Pb* liver development, since mice maintained under LP diet during infection presented lower *Pb* liver load *in vivo* 48 h after infection than mice under AP diet (**Figure S1F**). We thus assessed whether our protocol, in which LP diet is replaced by AP diet during the 5^th^ week of the experiment in order to minimize any effects on subsequent parasite development, did not impact on *Pb* infection. Indeed, mice that had been under LP or AP diet and were infected on day 35 presented similar parasite load in the liver 48 h after infection (**Figure 1F**). In addition, we found comparable blood parasitemia after infection (**Figure S1G**).

We have thus established a protocol of reduced dietary protein that impacts on the host’s liver metabolism and immune system, without affecting *Pb* development.

### Impact of Dietary Protein Reduction on the Circulating Immune Compartment Upon RAS Immunization

**Figure 2:**
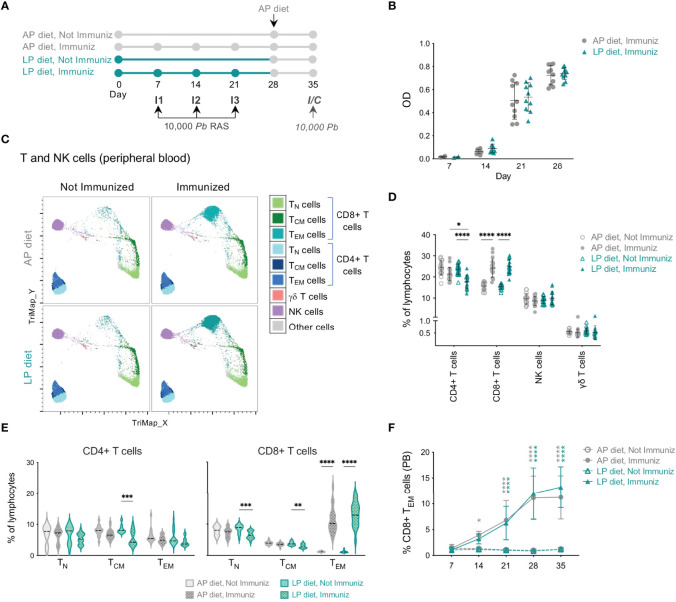
Impact of low protein diet during RAS immunization on the circulating immune response. **(A)** Protocol used in the study. The immunization schedule consisted of 3 immunizations (I1, I2, I3) with 10,000 *Pb*RAS under adequate (AP; 20%) or low (LP; 9%) protein diet, followed by one week under AP diet. Non-immunized and immunized mice were subsequently infected/challenged (I/C) on day 35. **(B)** Levels of anti-*Pb* sporozoite IgG in the plasma of mice immunized under AP or LP diet. **(C)** TriMap dimensionality reduction analysis of circulating immune populations (day 35) in concatenated data of non-immunized or RAS-immunized mice maintained under AP or LP diets, based on the following markers: CD3, CD4, CD8, CD44, CD62L, TCRγδ and NK1.1. **(D)** Frequency of CD4+, CD8+, γδ T and NK cells in the peripheral blood of non-immunized or RAS-immunized mice under AP or LP diet on the day of the infection/challenge (day 35). Each symbol represents a mouse (n = 15 to 20 per group). **(E)** Frequency of T CD4+ and CD8+ naïve (T_N_), central memory (T_CM_) or effector/effector memory (T_EM_) populations in mice under each condition on day 35 (n = 13-20). **(F)** Frequency of CD8+ T_EM_ in the peripheral blood (PB) of mice under each condition (n = 11-20 per group). The symbols represent statistically significant differences between non-immunized and RAS-immunized mice within AP (in grey) or LP (in blue) diet. Data are presented as mean ± SD and were compared using the Mann-Whitney test (two-sample data) or the Kruskal-Wallis test with Dunn’s multiple comparison post-test with selection of the following pairs: AP diet Not Immunized (NImm) vs. AP diet Immuniz (Imm), LP diet NImm vs. LP diet Imm, AP diet NImm vs. LP diet NImm and AP diet Imm vs. LP diet Imm (*P < 0,05; **P < 0,01; ***P < 0,001; ****P < 0,0001).

To study the impact of the diet during immunization on the host’s immune system, mice maintained under our established protocol of LP or AP diet were subjected to a prime-boost-boost vaccination regimen employing 10,000 *Pb* RAS per immunization (**Figure 2A**), previously reported as being highly protective against malaria (10). Of note, an increase in the expression of *srebf1* and *fas* was confirmed in all mice immunized under LP diet as compared to those under AP diet, on day 35 (**Figure S2**).

We first analyzed the humoral response against *Pb* sporozoites elicited by RAS immunization of mice under LP or AP diets. Mice immunized under LP diet revealed no differences in total anti-*Pb* sporozoite IgG antibody titers after each immunization relative to those maintained under AP diet (**Figure 2B**), indicating that the dietary protein reduction employed does not impair the host’s ability to mount a humoral response.

Alterations in the host’s immune system, and specifically in T and NK cell populations, which have well-established pathophysiological roles during *Plasmodium* infection (13, 36), were then examined in the blood of mice immunized with RAS under LP or AP diets using bidimensional hierarchical gating and TriMap dimensionality reduction. Two weeks after the 3^rd^ immunization (day 35) no differences were observed in the frequency or numbers of circulating NK or γδ T cells in immunized mice under either type of diet (**Figures 2C, D** and **Figure S3A**). In contrast, a clear increase in the frequency and number of circulating CD8+ T cells upon immunization of mice under either AP or LP diet was observed (**Figures 2C, D** and **Figure S3A**). This increase was mostly due to the early expansion of CD8+ T_EM_ cells upon immunization, which was similar in mice immunized under LP or AP diets (**Figures 2C, E** **, F** and **Figure S3B**). Specifically, all immunized animals presented an increase in the frequency of CD8+ T cells presenting an antigen-experienced effector phenotype, as indicated by the low levels of CD8α and CD127 of the expanded population (**Figure S3C, D**). Importantly, we also observed a significant decrease in the frequency of circulating CD4+ T cells in mice immunized under LP diet as compared to those under AP diet (**Figure 2D**).

Overall, we found that a reduction in dietary protein content during RAS immunization did not hamper the formation of a humoral response or the expansion of circulating CD8+ T_EM_ cells, but led to a decrease in the frequency of total CD4+ T cells in the blood.

### Impact of Dietary Protein Reduction on the Tissue Immune Compartment Upon RAS Immunization

**Figure 3:**
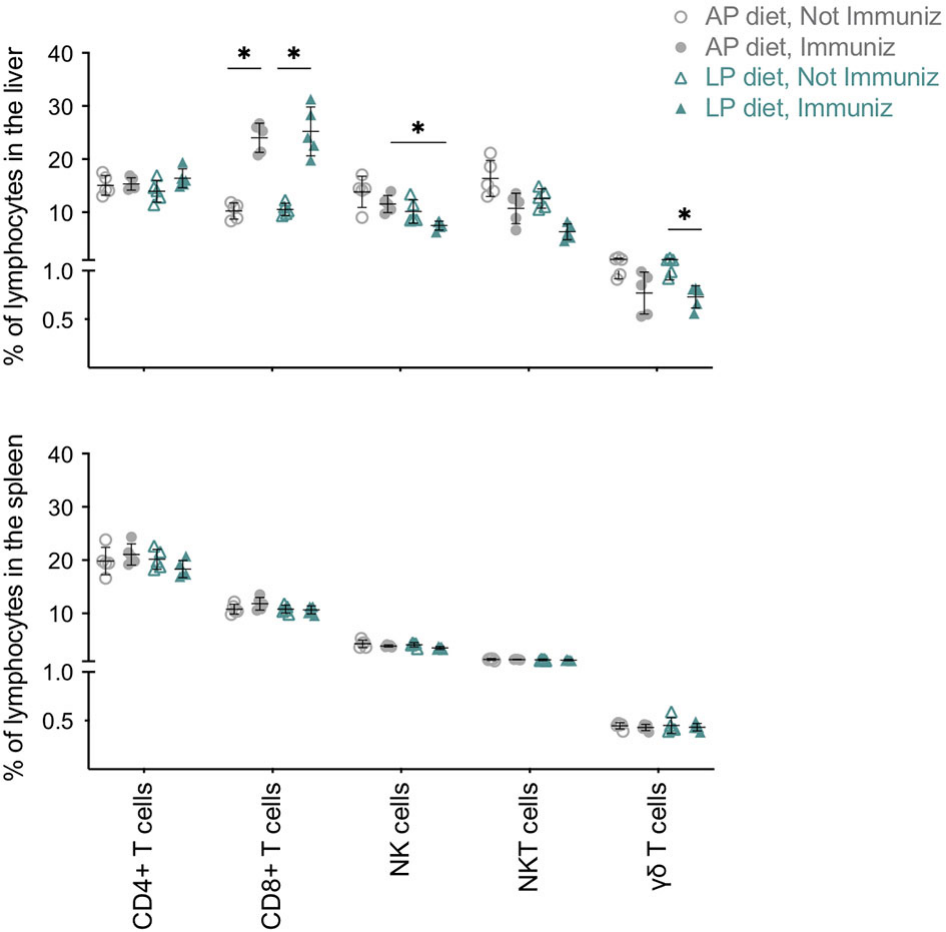
Impact of low protein diet during RAS immunization on the immune populations of the liver and spleen. Frequency of CD4+ T, CD8+ T, NKT, γδ T and NK cells in the liver and spleen of non-immunized or RAS-immunized mice under AP or LP diet on the day of infection/challenge (day 35). Data are presented as mean ± SD and were compared using the Kruskal-Wallis test with Dunn’s multiple comparison post-test, with selection of the following pairs: AP diet Not Immunized (NImm) vs. AP diet Immuniz (Imm), LP diet NImm vs. LP diet Imm, AP diet NImm vs. LP diet NImm and AP diet Imm vs. LP diet Imm (*P < 0,05).

We next analyzed the tissue-associated immune responses generated upon RAS immunization under the two diets. Overall, no diet- or immunization-associated differences in cell population frequencies or numbers were observed in the spleen on day 35 (**Figure 3** and **Figure S4A**). In contrast, in the livers of immunized mice we found a diet-related reduction in NK cell frequency and a similar trend in NKT cells (**Figure 3**). In addition, mice under LP diet had a decrease in their proportion of γδ T cells upon immunization (**Figure 3**). We also observed a significant expansion of CD8+ T cells in the liver of immunized mice, independently of the diet (**Figure 3** and **Figure S4A**), which positively correlated with the increase in CD8+ T cells found in the blood (**Figure S4B**).

**Figure 4:**
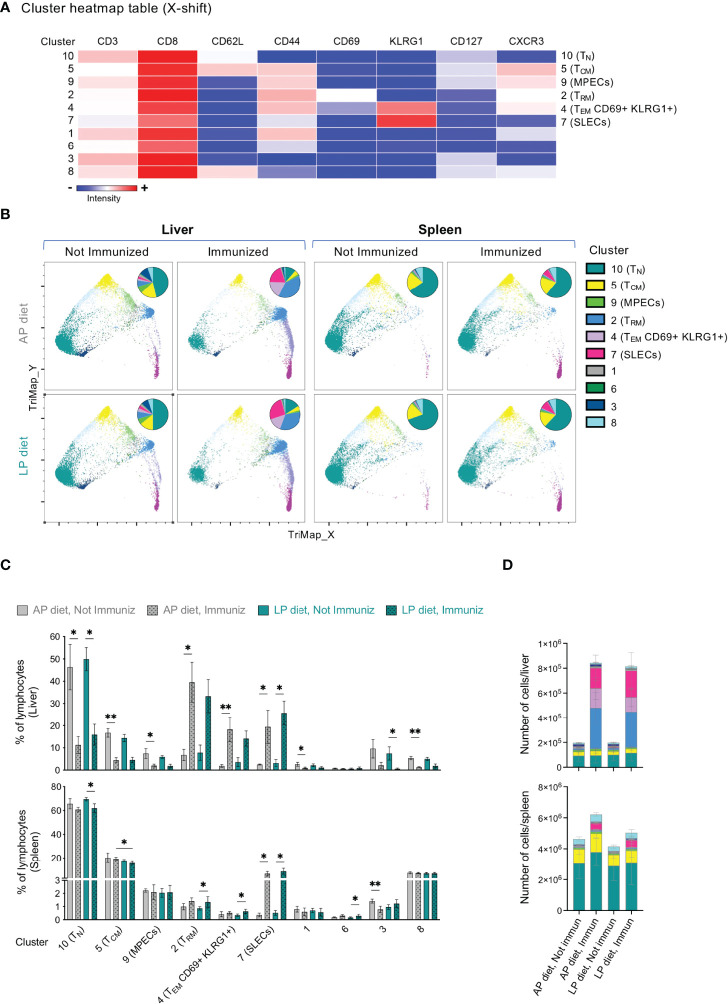
Clustering analysis of the CD8+ T cell pool in the liver and spleen of mice under AP or LP diet. **(A)** Heatmap table of the clusters identified by X-shift analysis of concatenated data from CD8+ T cells of the liver and the spleen of non-immunized or RAS-immunized mice under AP or LP diet on the day of the infection/challenge (day 35). T_N_: naïve T cells; T_CM_: central memory T cells; T_EM_: effector/effector memory T cells; T_RM_: resident memory T cells; SLECs: short-lived effector cells; MPECs: memory-precursor effector cells; KLRG1: killer cell lectin-like receptor subfamily G, member 1. **(B)** X-shift clustering analysis of concatenated data from the liver or spleen of mice under each condition. Data is presented using TriMap dimensionality reduction. The inserts in each plot represent the distribution of the clusters within the CD8+ T cell population of each condition. Frequency **(C)** or numbers **(D)** of each of the clusters identified in the X-shift analysis in **(A)** in the liver or spleen, for each condition. Data are presented as mean ± SD and were compared using the Kruskal-Wallis test with Dunn’s multiple comparison post-test, with selection of the following pairs: AP diet Not Immunized (NImm) vs. AP diet Immuniz (Imm), LP diet NImm vs. LP diet Imm, AP diet NImm vs. LP diet NImm and AP diet Imm vs. LP diet Imm (*P < 0,05; **P < 0,01).

Given the central role of CD8+ T cells in mediating protection upon RAS immunization, we further dissected the composition of the CD8+ T cell populations in the liver and spleen using in-depth unsupervised analysis of flow cytometry data. The X-shift algorithm (37) was applied to concatenated data sets comprising the CD8+ T cell populations of both the spleens and the livers of non-immunized and immunized mice under LP or AP diet on the day of infection/challenge (day 35), and the clustering analysis was visualized using TriMap dimensionality reduction (**Figures 4A, B**). Several clusters could be associated with previously described CD8+ T cell subpopulations based on the markers expressed (**Figure 4A**), including T_RM_ cells (cluster 2, CD62L–CD44+CD69+KLRG1–CXCR3+; KLRG1: killer cell lectin-like receptor subfamily G, member 1), memory-precursor effector cells (MPECs; cluster 9, CD62L–CD44+KLRG1–CD127+) and short-lived effector cells (SLECs; cluster 7, CD62L–CD44+KLRG1+CD127–), which are associated with distinct function, migratory properties, survival and memory potential of effector CD8+ T cells (38, 39).

Analysis of splenic CD8+ T cell cluster frequency (**Figures 4B, C**) and numbers (**Figure 4D** and **Figure S5**) upon immunization revealed a significant decrease in the proportion of CD8+ T_CM_ cells (cluster 5, CD62L+CD44+) in mice immunized under LP as compared to those under AP diet. In addition, a significant increase in splenic and liver SLECs (cluster 7) upon immunization was observed irrespective of the diet (**Figures 4B–D** and **Figure S5A**).

As expected, mice under AP diet displayed a significant increase in the frequency and numbers of hepatic CD8+ T_RM_ cells (cluster 2) upon immunization, a trend that was also observed, though not statistically significant, in mice under LP diet (**Figures 4B–D** and **Figure S5A**). Interestingly, we further observed an immunization-associated increase in a population of T_EM_ cells co-expressing CD69 and KLRG1 (cluster 4; CD62L–CD44+CD69+KLRG1+CXCR3+), which appears to present an intermediate profile between T_RM_ and SLECs (**Figure 4B**). Joint analysis of clusters 2 and 4 yielded similar results as those obtained for either cluster alone, including the absence of statistically significant diet-related differences (**Figure S5B**). Finally, we found a decrease in the frequency of the hepatic CD8+ T cell clusters corresponding to T_N_ (cluster 10, CD62L+CD44–), T_CM_ (cluster 5, CD62L+CD44+) and MPECs (cluster 9) upon immunization with either diet (**Figures 4B–D** and **Figure S5A**). Of note, although these alterations were mostly related to the immunization protocol *per se* and not to the diet, they often reached statistical significance in mice immunized under AP diet, but not in mice immunized under LP diet.

In conclusion, we found that LP diet during RAS immunization was associated with a decrease in the frequency of NK cells in the liver and of CD8+ T_CM_ cells in the spleen as compared to immunization under AP diet, but did not drastically affect the tissue dynamics of the hepatic CD8+ T cell pool.

### Efficacy of RAS Vaccination Under Reduced Dietary Protein

**Figure 5:**
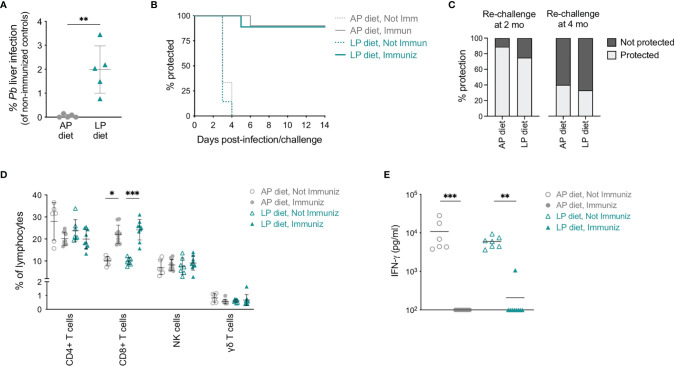
Efficacy of RAS immunization under low protein diet. **(A)** Relative parasite liver load (based on *Pb* 18S rRNA expression and compared to the corresponding non-immunized group) 48 h post-challenge of mice immunized under AP or LP diet. **(B)** Protection of non-immunized and RAS-immunized mice under AP or LP diet upon infection/challenge, as assessed by blood parasitemia (n = 6-10 mice per group). **(C)** Protection of RAS-immunized mice under AP or LP diet when re-challenged 2 or 4 months (mo) after the first challenge. Mice without detectable blood parasitemia until day 14 were considered protected (n = 3-9 mice per group). **(D)** Frequency of CD4+ T, CD8+ T, γδ T and NK cells in the peripheral blood of non-immunized or RAS-immunized mice under AP or LP diet, 5 days after infection/challenge. **(E)** Levels of IFN- γ in the plasma of non-immunized or RAS-immunized mice under AP or LP diet, 5 days after infection/challenge. Data are presented as mean ± SD and were compared using the Mann-Whitney test (two-sample data), or the Kruskal-Wallis test with Dunn’s multiple comparison post-test with selection of the following pairs: AP diet Not Immunized (NImm) vs. AP diet Immuniz (Imm), LP diet NImm vs. LP diet Imm, AP diet NImm vs. LP diet NImm and AP diet Imm vs. LP diet Imm (*P < 0,05; **P < 0,01; ***P < 0,001).

To understand whether a reduced protein intake would have an impact on the protection conferred by RAS immunization, mice previously immunized under AP or LP diets were challenged by intravenous injection of 10,000 infectious *Pb* sporozoites on day 35 (see **Figure 2A**), and parasite load in their livers was assessed 48 h later. Notably, higher liver infection loads were observed in mice immunized under LP diet than in their counterparts under AP diet (**Figure 5A**). Nevertheless, no significant differences were observed in the prepatent period or in protection against blood stage infection between mice immunized under AP or LP diets (**Figure 5B**), indicating that reduction in dietary protein during immunization does not significantly alter the outcome of vaccination upon challenge. Of note, non-immunized mice lost around 10% of their body weight between days 5 and 7 after infection, irrespective of the diet employed (**Figure S6A**). In addition, no differences were observed in long-term protection when mice immunized under either diet were re-challenged with 10,000 infectious *Pb* sporozoites after up to 4 months (**Figure 5C**).

Comparison of the circulating immune responses generated upon infection/challenge revealed a sharp decrease in the numbers of leukocytes in the blood of non-immunized, but not of immunized mice, between days 2 and 5 after infection, independently of the diet (**Figure S6B**). This decrease resulted in low numbers of all circulating immune populations analyzed in non-immunized mice (**Figure S6C**). In terms of frequency, while CD8+ T cells remained significantly increased in immunized as compared to non-immunized mice, no diet-related differences were found (**Figure 5D**).

Finally, we compared the levels of IFN-γ in the blood of non-immunized and RAS-immunized mice under AP or LP diet. In agreement with what was previously reported (40), high levels of plasma IFN-γ were found in non-immunized mice at day 5 post-infection (**Figure 5E**). In contrast, and in line with the observed protection from infection, RAS-immunized mice presented low to undetectable IFN-γ levels upon challenge (**Figure 5E**). Importantly, the levels of IFN-γ were not associated with the dietary protein content during the immunization period.

In conclusion, we found that, although low dietary protein intake during RAS immunization is associated with an increase in the parasite liver load, it does not preclude the formation of a protective immune response against *Plasmodium* infection.

## Discussion

Based on the effect of malnutrition on the immune response (18, 41) and on the disparity of results obtained in clinical trials for malaria vaccine candidates in high-income versus endemic countries (15, 16), we hypothesized that inadequate protein intake could have an impact on the generation of an efficient response to anti-malarial vaccination. Since the protein consumption of populations living in malaria-endemic regions may vary depending on their area of residence and economic status, and even throughout the different seasons of the year, our experimental dietary conditions were selected based on the mean consumption data provided by the United Nations’ Food and Agriculture Organization (17, 19, 20).

We established a model for RAS immunization under a low protein diet and a subsequent challenge under protein-sufficient conditions that alters the nutritional status of the host, without affecting parasite development. We showed that our newly-established protocol successfully accomplished the proposed objectives, since: i) mice under LP diet gained less weight and consumed more water than their counterparts receiving AP diet, in line with previous reports involving reduced dietary protein (22, 23, 42); ii) the livers of mice under LP diet showed increased expression of lipogenesis-related genes, in agreement with the association of low dietary protein with hepatic lipid accumulation and increased risk of fatty liver disease (43, 44); iii) following challenge, *Pb* liver load and blood parasitemia were equivalent in non-immunized mice maintained under LP or AP diet.

Despite the significant impact of dietary protein reduction on body weight and the diet-induced differences in lipogenesis-related hepatic genes observed, we did not find significant alterations in plasma metabolites of mice under LP diet, when compared with their AP counterparts. Unaltered plasma protein or albumin had been previously reported in mice and rats under low protein diets (42, 45). Of note, the similar levels of plasma alanine aminotransferase (ALT), an indicator of hepatic injury, in mice under LP and AP diets indicates that the reduced protein levels used in our protocol, which do not correspond to a complete absence of dietary protein, did not result in liver damage, as confirmed by histological analysis (data not shown).

Severe dietary protein reduction, such as that used in PEM models (usually less than 3% of protein), reportedly leads to spleen and thymus atrophy and to a decrease in B and T cell numbers (21, 23–25, 46). The less pronounced impact of the diet on the immune system observed in our model, which included a decrease in the proportion of T_EM_ within circulating CD8+ T cells, was most probably associated with the milder reduction in dietary protein content employed. Of note, cellular function and homeostatic fitness were not analyzed in this study, nor were other innate immune populations, which could reveal important unaddressed modifications.

The lower frequency of circulating CD4+ T cells in mice immunized under LP as compared to AP diet suggests an effect of protein reduction on the CD4+ T cell compartment. Indeed, previous studies have indicated an important role for dietary protein in memory CD4+ T cell function in mice (47), and of L-arginine in regulating metabolic pathways involved in CD4+ T cell memory survival and differentiation in humans (48). Of note, it is unlikely that follicular T helper cells (T_FH_), a specialized subset that regulates the development of antigen-specific B cell immunity (13), were severely affected by the low protein levels during immunization, since no diet-related differences were found in the humoral response elicited by vaccination.

RAS immunization under LP diet also resulted in a reduced proportion of hepatic NK and NKT cells, compared to AP diet. Since both of these subsets were shown to contribute to IFN-γ production during *Plasmodium* infection, with NK cells being one of its earliest sources during the liver stage of the parasite’s life cycle (49) and NKT cells being able to inhibit parasite growth in hepatocytes in a partially IFN-γ-dependent manner (50), we may speculate that this decrease could have contributed to the higher liver parasite load observed in mice immunized under LP diet compared to their AP counterparts.

Given the critical role of CD8+ T cells in malaria immunity (13, 14), understanding their profile and dynamics is important for the design of improved vaccination strategies. Severe dietary protein reduction reportedly has a detrimental effect on CD8+ T cell memory, and was associated with low levels of antigen-specific memory CD8+ T cells, their decreased homeostatic proliferation and impairment of recall responses upon viral infection (23, 24). We found that the profile of memory CD8+ T cells generated during RAS immunization was considerably robust and minimally affected by the LP diet employed in our protocol. In addition, our data on the vigorous expansion of a circulating CD44+ T cell population expressing low levels of CD8α (51, 52) hints at the generation of a comparable *Plasmodium*-specific pool in mice immunized under LP or AP diet. Although the antigen specificity of CD8+ T cells was not directly assessed in this study, the similar protection against a later *Plasmodium* challenge observed in mice immunized under either diet also supports the absence of significant differences at that level. Moreover, unbiased clustering analysis of CD8+ T cells from the spleen and liver of non-immunized or RAS-immunized mice under each diet showed that most of the alterations observed were not related to protein intake, but rather to the immunization process. Importantly, no statistically significant diet-related differences were observed in the generation of liver CD8+ T_RM_ cells, which are considered part of the first line of defense that prevents dissemination of invading pathogens, and crucial for RAS-induced protection (11–14). Of note, there was accumulation of a CD8+ T cell population with a short-lived and cytotoxic SLEC phenotype in both the spleen and liver, while T_CM_ and MPECs, which display increased survival and developmental plasticity toward the memory phenotype (38), were unaltered or decreased, with potential implications for the immune response to subsequent infections. Ultimately, the similar outcome of *Pb* challenge up to more than 4 months after immunization, regardless of the protein intake during immunization, suggests that any diet-related alterations that may have occurred in the CD8+ T cell pool did not differentially impact on the efficacy of the immune response to infection.

Importantly, our X-shift analysis of the CD8+ T cell pool in non-immunized and RAS-immunized mice identified the expansion of two putative liver-resident CD69+ CD8+ T cell populations, which could be distinguished based on the higher (cluster 4) or lower (cluster 2) levels of KLRG1. Our data raises the prospect of further investigating the functional potential of each subpopulation independently. In addition, and in light of the recently reported existence of liver CD69+ CD8+ T cell populations derived from either KLRG1– or “exKLRG1” (long-lived cells derived from KLRG1+ effector CD8+ T cells that lost KLRG1) memory cells (53), future studies should also address the precursor-progeny relationship of these populations.

Since malnutrition can assume multiple forms and may be related to alterations in several types of nutrients, our model can be employed to study the impact of other nutritional imbalances, individually or in combination, on malaria vaccination. This may include, for example, micronutrients like vitamin A or zinc, whose insufficiency has been shown to lead to a decreased ability to mount efficient memory immune responses (18, 19, 21).

In conclusion, we have established a model that allows the investigation of the impact of the nutritional status of the host on the immunogenicity and efficacy of RAS immunization, with minimal impact on the parasite. We show that reduced protein intake, comparable to that found in malaria-endemic regions, did not impair the immunogenicity or protective efficacy of RAS vaccination, corroborating the use of this strategy in low dietary protein settings. Importantly, our model can be extended to investigate other nutrient imbalances, as well as to additional immunization strategies that do not rely on parasite development, such as subunit vaccine candidates, providing a basis for potential rationales of translational interventions that improve vaccine efficacy in malnourished individuals.

## Data Availability Statement

The original contributions presented in the study are included in the article/**Supplementary Material**. Further inquiries can be directed to the corresponding authors.

## Ethics Statement

The animal study was reviewed and approved by iMM-JLA’s animal ethics committee (ORBEA). All experimental animal work was performed in strict compliance with the guidelines of the Federation of European Laboratory Animal Science Associations (FELASA).

## Author Contributions

HN-C and MP designed the study. HN-C, DM, CR, and AM performed research and analyzed the data. HN-C, AM, and MP discussed the results. HN-C wrote the paper. All authors contributed to the article, reviewed the manuscript and approved the submitted version.

## Funding

This work was funded by Grant HR21-00848 of the ”la Caixa” Foundation and Grant TC269 of the GSK "Open Lab" Foundation, awarded to MP. HNC, AMM and MP received funding from Fundação para a Ciência e Tecnologia (references DL57/2016/CP1451/CT0011, DL57/2016/CP1451/CT0005 and CEECIND/03539/2017, respectively).

## Conflict of Interest

The authors declare that the research was conducted in the absence of any commercial or financial relationships that could be construed as a potential conflict of interest.

## Publisher’s Note

All claims expressed in this article are solely those of the authors and do not necessarily represent those of their affiliated organizations, or those of the publisher, the editors and the reviewers. Any product that may be evaluated in this article, or claim that may be made by its manufacturer, is not guaranteed or endorsed by the publisher.
