# Supplementary material for: Impact of Dietary Protein Restriction on the Immunogenicity and Efficacy of Whole-Sporozoite Malaria Vaccination
Source: Front Immunol. 2022 Apr 21;13:869757. doi: 10.3389/fimmu.2022.869757 (PMC9070679; doi:10.3389/fimmu.2022.869757)
Supplement: Supplementary file 1 [file DataSheet_1.pdf]

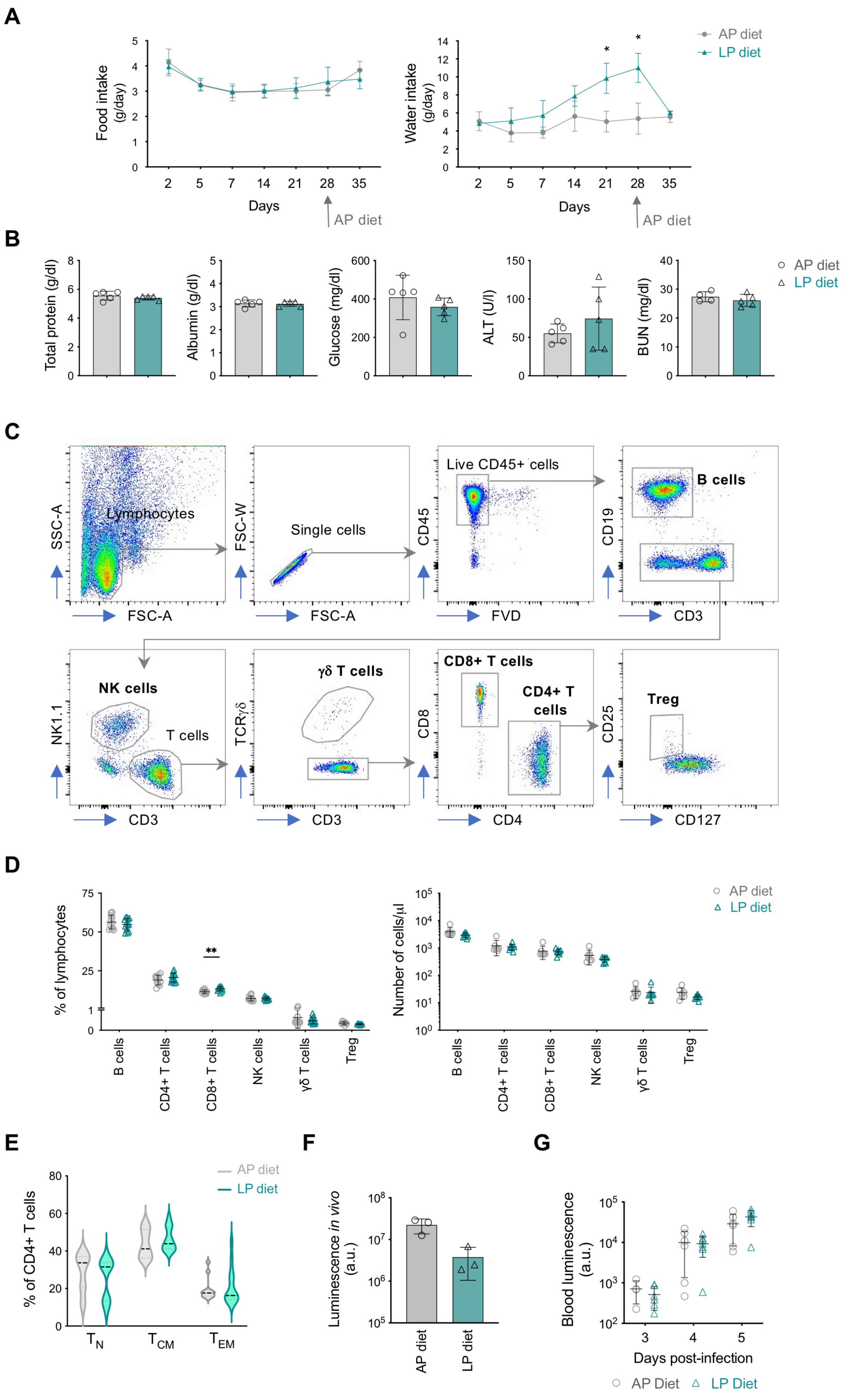

Fig. S1: Optimization of a protocol of low protein diet that impacts the host but not parasite development. (A) Food and water consumption per mouse under AP or LP diet. Results were calculated from 3-4 cages of 2-3 independent experiments. (B) Quantification of the levels of metabolites (total protein, albumin, glucose, alanine aminotransferase, ALT, and blood urea nitrogen, BUN) in the plasma of mice under adequate (AP, 20%) or low (LP, 9%) protein diet on day 35. (C) Representative gating strategy applied for the flow cytometric analysis of circulating immune populations. FVD: fixable

viability dye. (D) Frequency and number of cells of the immune populations defined in (C) in the peripheral blood of mice under AP or LP diet, on day 28. Each symbol represents a mouse (n=11-13 per group). (E) Violin plots of the frequency of naïve (T_N), central memory (T_{CM}) and effector/effector memory (T_{EM}) populations within CD4+ T cells in mice maintained under AP or LP diet, on day 28 (n=11-13 per group). (F) Liver parasite load (as measured by luminescence *in vivo*) of mice maintained under AP or LP diet for 2 weeks before infection with 30,000 *Pb* sporozoites, at 48 hours post-infection. (G) Blood parasitemia (as measured by luminescence) of mice maintained under AP or LP diet and infected with 10,000 *Pb* sporozoites on day 35. Data are presented as mean ± SD and were compared using the Mann-Whitney test (*: P<0,05; **: P<0,01; ***: P<0,001; ****: P<0,0001).

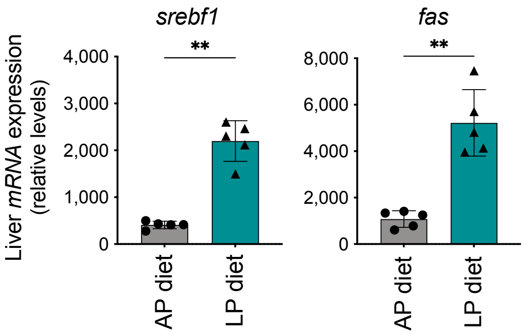

Fig. S2: Validation of the effect of the low protein (LP) diet on liver metabolism of immunized mice. Plots represent the hepatic expression of *srebf1* and *fas* on day 35 in mice maintained under adequate protein (AP) or LP diet. Values were normalized to *hprt* expression. Data are presented as mean ± SD and were compared using the Mann-Whitney test (*: P<0,05; **: P<0,01; ***: P<0,001; ****: P<0,0001).

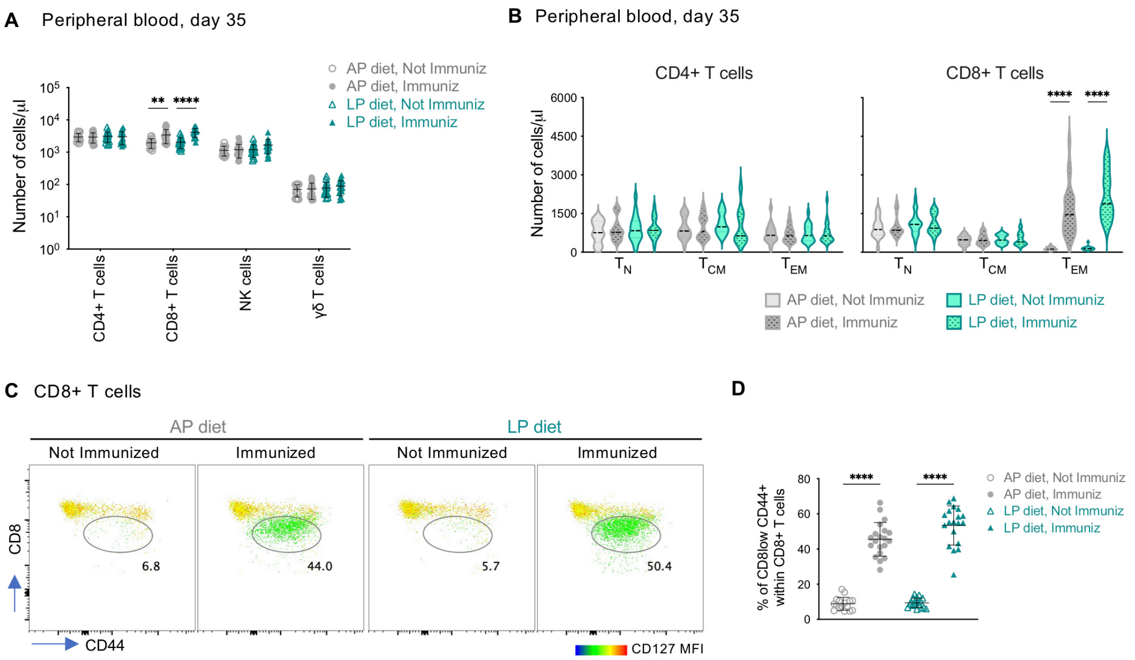

Fig. S3: Impact of low protein diet during RAS immunization on the circulating immune response. (A) Number of CD4+ T, CD8+ T, γδ T and NK cells in the peripheral blood of non-immunized or RAS-immunized mice under AP or LP diet on the day of the infection/challenge (day 35). Each symbol represents a mouse (n=15 to 20 per group). (B) Number of T CD4+ and CD8+ naïve (T_N), central memory (T_{CM}) or effector/effector memory (T_{EM}) cells in the peripheral blood of mice under each condition on day 35 (n=13-20). (C) Representative heatmap statistic plots of the expression of CD8α and CD44 on CD8+ T cells from the peripheral blood of non-immunized or RAS-immunized mice under AP or LP diet on day 35. The median fluorescence intensity (MFI) of CD127 is represented by the heatmap. (D) Quantification of the frequency of CD8lowCD44+ T cells, as gated in (C), within CD8+ T cells. Each symbol represents a mouse (n=16 to 20 per group). Data are presented as mean ± SD and were compared using the Kruskal-Wallis test with Dunn's multiple comparison post-test, with selection of the following pairs: AP diet Not Immunized (NImm) vs AP diet Immuniz (Imm), LP diet NImm vs LP diet Imm, AP diet NImm vs LP diet NImm and AP diet Imm vs LP diet Imm (*: P<0,05; **: P<0,01; ***: P<0,001; ****: P<0,0001).

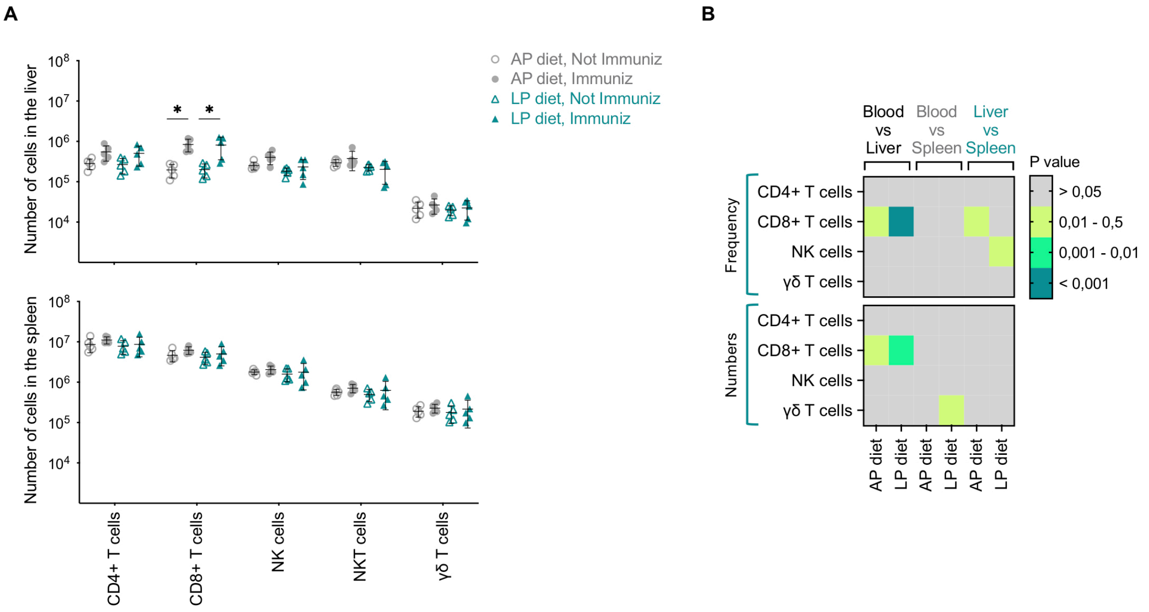

Fig. S4: Impact of low protein diet during RAS immunization on the immune populations of the liver and spleen. (A) Number of CD4+ T, CD8+ T, γδ T, NK and NKT cells in the liver and spleen of non-immunized or RAS-immunized mice under AP or LP diet on the day of infection/challenge (day 35). Data are presented as mean ± SD and were compared using the Kruskal-Wallis test with Dunn's multiple comparison post-test, with selection of the following pairs: AP diet Not Immunized (NImm) vs AP diet Immuniz (Imm), LP diet NImm vs LP diet Imm, AP diet NImm vs LP diet NImm and AP diet Imm vs LP diet Imm (*: P<0,05; **: P<0,01; ***: P<0,001; ****: P<0,0001). (B) Heatmap of the correlations (all positive) in the frequency (top graph) or numbers (bottom graph) of each cellular subset in the peripheral blood vs liver or vs spleen, or in liver vs spleen, in all mice under AP or LP diet on day 35 (n=10).

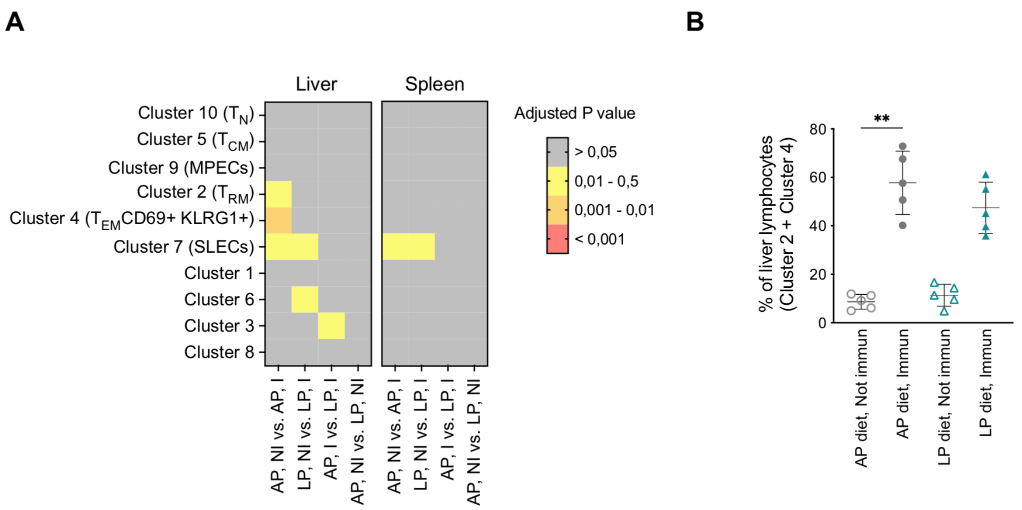

Fig. S5: Clustering analysis of the CD8+ T cell pool in the liver and spleen of mice under AP or LP diet. (A) Heatmap of the statistical analysis performed for Fig. 4D, on the number of cells within each cluster and in each condition per organ. (B) Frequency of clusters 2 + 4, representing total CD69+ CD8+ T cells, within liver leukocytes. Data are presented as mean ± SD and were compared using the Kruskal-Wallis test with Dunn's multiple comparison post-test, with selection of the following pairs: AP diet Not Immunized (AP NI) vs AP diet Immuniz (AP I), LP NI vs LP I, AP I vs LP I and AP NI vs LP NI.

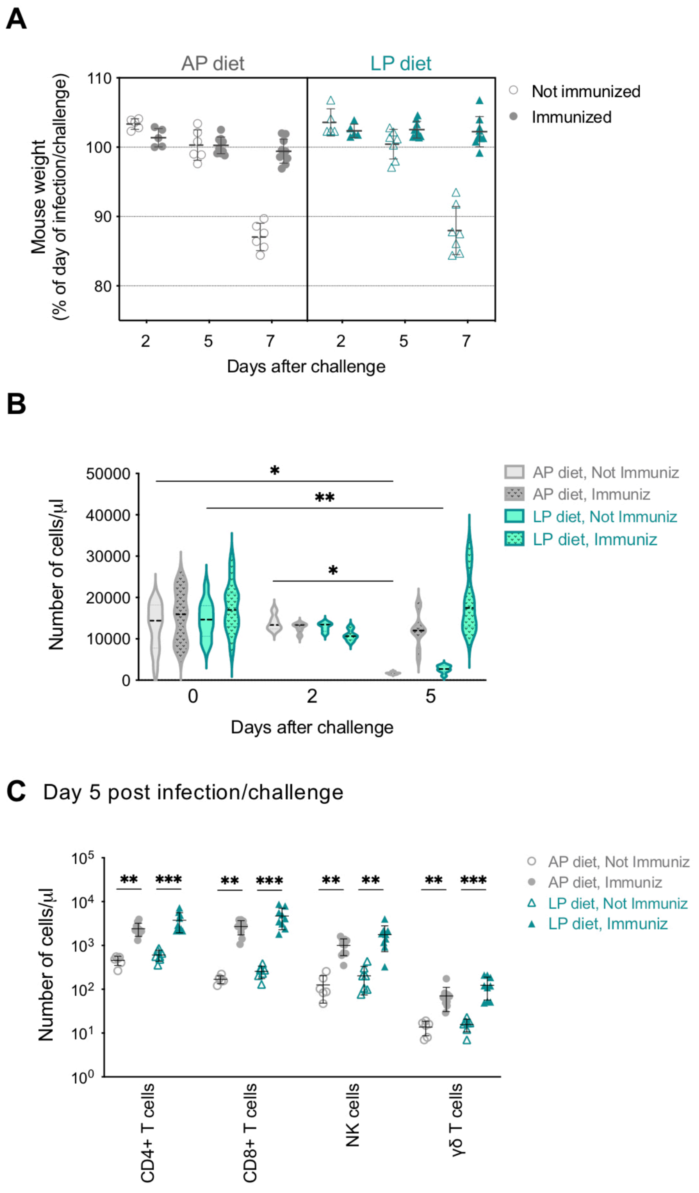

Fig. S6: Efficacy of RAS immunization under low protein diet. (A) Alteration in the weight of non-immunized and RAS-immunized mice under AP or LP diet after infection/challenge. (B) Number of cells in the blood of non-immunized and RAS-immunized mice under AP or LP diet after infection/challenge (n=5-11). (C) Number of CD4+ T, CD8+ T, γδ T and NK cells in the blood of non-immunized and RAS-immunized mice under AP or LP diet on day 5 post-infection/challenge (n=5-11). Data are presented as mean ± SD and were compared using the Kruskal-Wallis test with Dunn's multiple comparison post-test, with selection of the following pairs: AP diet Not Immunized (NImm) vs AP diet Immuniz (Imm), LP diet NImm vs LP diet Imm, AP diet NImm vs LP diet NImm and AP diet Imm vs LP diet Imm (*: P<0,05; **: P<0,01; ***: P<0,001; ****: P<0,0001).
